# Open-source pre-clinical image segmentation: mouse cardiac magnetic resonance imaging datasets with a deep learning segmentation framework

**DOI:** 10.1016/j.jocmr.2026.102706

**Published:** 2026-02-17

**Authors:** Wan Shah, Daniel J. Stuckey, Tina Yao, Mark Wrobel, Emily Deniszczyc, Zhiping Feng, Stephanie Anderson, Ruaraidh Campbell, Vivek Muthurangu, Jennifer Steeden

**Affiliations:** a UCL Centre for Translational Cardiovascular Imaging, Institute of Cardiovascular Science, University College London, London, UK; b UCL Centre for Advanced Biomedical Imaging, University College London, London, UK; c Department of Physiology, Anatomy and Genetics, University of Oxford, Oxford, UK

**Keywords:** Pre-clinical, Segmentation, Deep Learning, Open-Source

## Abstract

**Background:**

Longitudinal cardiovascular magnetic resonance (CMR) in small animal models is essential for studying cardiovascular disease mechanisms and assessing new therapies. However, conventional manual segmentation of ventricular structures from cine short-axis images is slow, labor-intensive, and subject to substantial observer variability, limiting reproducibility in large-scale pre-clinical studies. While deep learning (DL) segmentation has become standard in human CMR, existing models do not generalize to pre-clinical datasets. Current mouse-specific approaches have been constrained by small private cohorts, where the DL segmentation models are not published.

**Methods:**

Here, we present the first publicly available pre-clinical CMR dataset, along with an open-source DL segmentation model and a web-based interface for easy deployment.

The dataset comprises complete cine short-axis CMR images from 130 mice with diverse phenotypes, acquired at 9.4T. The dataset also contains expert manual segmentations of left ventricular (LV) blood pool and myocardium at end-diastole, end-systole, as well as additional timeframes with artifacts to improve robustness.

Using this resource, we developed an open-source DL segmentation model based on the UNet3+ architecture. The model was evaluated against an independent internal test dataset (9.4T, n = 25 mice), as well as two external test datasets (n = 15 mice at 7T, and n = 10 mice at 11.7T) and compared to expert manual segmentations in terms of Dice and functional parameters.

**Results:**

At inference, the DL segmentation model took <20 ms per 2D image, enabling complete cine stack segmentation in ∼4.6 s—over 6,000-fold speed-up over manual analysis. It achieved high segmentation accuracy in both the blood pool and myocardium in all test datasets (overall Dice ≥ 0.91). In addition, functional parameters showed excellent agreement with manual ground-truth measurements (intraclass correlation coefficient (ICC) ≥ 0.89 for the internal test dataset, and ICC > 0.85 for the external test datasets), with ICC values comparable to human inter-observer variability.

**Conclusion:**

This work provides the first open-access mouse cardiac MRI dataset, an open-source DL segmentation model, and an accessible inference platform. These tools establish a benchmark for pre-clinical CMR, enabling reproducible, scalable, and community-driven development of DL methods to accelerate cardiovascular research.

## Graphical abstract

**Figure:**
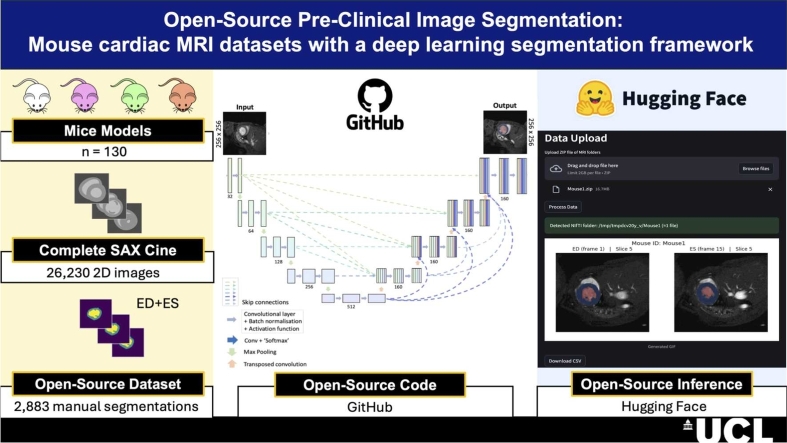

## 1 Introduction

Longitudinal monitoring of cardiovascular function in pre-clinical small animal models is essential for understanding disease progression and evaluating new therapeutic strategies. Cardiovascular magnetic resonance (CMR) has become the gold-standard method for assessing cardiac function, allowing non-invasive quantification of ventricular volumes, ejection fraction, and myocardial mass. These metrics are conventionally derived from segmentation of two-dimensional (2D) multi-slice short-axis (SAX) cine images, at end-diastole (ED) and end-systole (ES). However, current manual segmentation approaches are time-consuming and labor-intensive, which is particularly problematic when performing therapeutic trials with large numbers of animals. In addition, manual segmentations are prone to high levels of intra- and inter-observer variability [1], [2], [3], [4], which limits reproducibility. Therefore, it is necessary to develop rapid and reproducible solutions for analysis of large, longitudinal pre-clinical studies.

In human studies, many tools have been developed to automate segmentation of cardiac MRI data and improve reproducibility, with current state-of-the-art methods relying on deep learning (DL) [5], [6], [7]. These methods have been shown to be rapid and accurate, as well as being robust to multi-site and multi-vendor data in large cohort studies [8], [9]. However, DL segmentation models trained using human cardiac MRI data perform poorly when applied to pre-clinical CMR images.

Pre-clinical cardiovascular research commonly uses rodent models, with recent papers showing DL models for segmentation of the ventricles from CMR [10], [11], [12]. In rats, these DL models have been trained with large amounts of data (from >150 rats) and can achieve high segmentation accuracy, with low bias in clinical metrics and lower variability than seen between human observers. However, mice are the most widely used species for animal studies of cardiovascular disease [13], as they have a short gestation time, low maintenance/housing costs and can be genetically modified to enable the investigation into mechanisms underlying pathogenesis. Current DL models for segmentation of mouse CMR data have been trained with low numbers of datasets (from < 21 mice) [14], [15], [16], limiting their accuracy and generalizability. This is problematic as mouse images have inherently lower signal-to-noise (SNR) and more artifacts, due to higher heart rates and smaller heart size of mice. In addition, all current pre-clinical DL segmentation methods rely exclusively on private datasets for training, making comparison of new methodologies difficult, and the current DL segmentation models are not published, meaning they cannot be used by the wider scientific community. To truly harness the full potential of DL segmentation models in mice, large publicly available datasets, as well as open-source DL models are needed.

Therefore, this study aims to (i) Create the first publicly available database of pre-clinical mouse cardiac MR images (acquired at 9.4T) with expert contours; (ii) Develop an open-source DL segmentation model, optimized for left ventricular (LV) blood pool and myocardial segmentation in this pre-clinical mouse dataset; (iii) Validate the open-source DL model in terms of segmentation accuracy and conventional ventricular volumes in mice; (iv) Test the generalizability of the open-source DL model on two external pre-clinical mouse datasets (acquired at a different site and at different field-strengths; 7T and 11.7T, using different protocols, and including a different scanner vendor); (v) Provide a web-based interface for easy deployment of this segmentation model.

## 2 Methods

### 2.1 . Publicly available pre-clinical cardiac MRI dataset

#### 2.1.1 Dataset characteristics

The dataset, which we have made publicly available, includes 130 mice imaged at 9.4T between June 2012 and December 2019. Each mouse consists of a complete short-axis cine stack, with ∼9 slices (range 8–11) and ∼22 cardiac timeframes (range 17–36), resulting in a total of 26,230 2D images. The dataset encompasses a broad range of myocardial phenotypes as follows: (i) Controls (n = 39); (ii) Myocardial infarction with grafting of a biomaterial patch (n = 11); (iii) Transgenic model of dilated cardiomyopathy (n = 51); and (iv) Transverse aortic constriction model (n = 29).

All experiments were conducted in full compliance with the European Union Directive 2010/63/European Union and the UK Animals Scientific Procedure Act 1986. Experimental protocols were approved by the UK Home Office under Project Licenses 70_8709 and PP1692884, and by the Animal Welfare and Ethical Review Body at University College London (UCL).

#### 2.1.2 Imaging protocol

The dataset was acquired on a 9.4T small-animal Agilent MRI system (Agilent Technologies, Santa Clara, California) equipped with 1000 mT/m gradient inserts and a 39 mm volume resonator RF coil (RAPID Biomedical, Rimpar, Germany). A small animal physiological monitoring system (SA Instruments, Stony Brook, New York) was used to maintain depth of anesthesia and animal physiology. The electrocardiogram (ECG) trace was recorded using 3-lead subcutaneous electrodes, respiration rate was measured by a pressure sensitive balloon and internal temperature by rectal thermometer. Animals were anaesthetized under a mixture of 1%–2% isoflurane in oxygen.

For each mouse, cardiac-gated, spoiled gradient echo (GRE) cine MRI was acquired as described in [17]. A stack of 8–11 contiguous slices was used to ensure full coverage of the left ventricle. Prospective ECG triggering was used to synchronize image acquisition with the cardiac cycle, and respiratory gating was used to remove breathing motion. The imaging parameters were: slice thickness = 1.0 mm; TE/TR = 1.2/5.0 ms; flip angle = ∼16°; acquisition matrix size = 128 × 128; signal averages = 3, interpolation factor = 2.0; interpolated pixel size = 0.1 × 0.1 mm. The temporal resolution was 5.0 ms, resulting in 17–36 timeframes per cardiac cycle (depending on heart rate).

#### 2.1.3 Ground-truth segmentation

Manual segmentation of the LV blood pool and myocardium was performed on all slices, at end-diastole and end-systole, by one observer (W.N.H., 2 years’ experience) using Horos version 3.3.6 (The Horos Project, https://horosproject.org). Trabeculae and papillary muscles were included in the myocardial mass. The contours were checked by two independent experts (D.S. and V.M., 22 and 23 years’ experience, respectively) who had to reach consensus in case of discordance.

It was observed that the cine images often contain inflow-related artifacts in early-diastolic and mid-systolic timeframes. Therefore, to improve model robustness and enable the use of the DL segmentation model throughout the cardiac cycle, additional timeframes containing inflow artifact were also segmented. Specifically, segmentations were performed in all slices for 44 additional cardiac timeframes (taken from four mice). This resulted in datasets from 304 cardiac timeframes (each containing all slices of the SAX), i.e., 2883 2D images, with ground-truth manual LV blood pool and myocardial segmentations.

#### 2.1.4 Data format and availability

The dataset is shared in HDF5 format, with one.h5 file per mouse. Each HDF5 file contains the full cine SAX dataset and the ground-truth manual segmentations for the LV blood pool and myocardium. For each mouse, the imaging data (h5 dataset name *‘Images’*) is of size: *matrix_size_x × matrix_size_y × number_of_slices × number_of_time_frames*, where the matrix size is 256 × 256, the number of slices is in the range 8–11 and the number of timeframes is in the range 17–36. The segmentation data (h5 dataset name *‘Masks’*) is the same size as the image data, with values in the range 0–2, representing voxels belonging to the background (0), the LV myocardium (1), and LV blood pool (2). A third parameter is given in the HDF5 file, which identifies the cardiac timeframes which contain ground-truth manual segmentation (h5 dataset name *‘Frames_with_ROI'*). Cardiac timeframes which do not contain manual segmentation consist of only zeros in the ‘*Masks’* data.

The dataset for all 130 mice is available at: https://github.com/mrphys/Open-Source_Pre-Clinical_Segmentation.git.

### 2.2 Open-source pre-clinical segmentation model

**Fig. 1:**
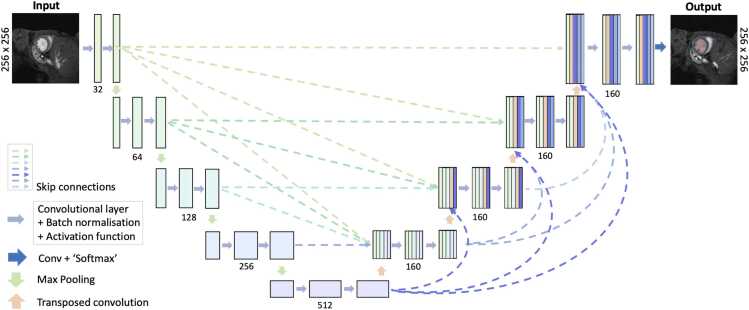
UNet3+ structure used in our open-source deep learning model. The DL model provides fully automated segmentation of LV blood pool and myocardial per 2D image. The numbers at each level represent the number of channels. *DL* deep learning, *LV* left ventricular, *2D* two-dimensional

A number of network architectures have been demonstrated for medical image segmentation, including the UNet [18], nnUNet [19] and TransUNet [20]. In this study, we developed an open-source 2D DL segmentation model based on the UNet3+ architecture [21]. This is a network architecture which we have previously shown to achieve very good segmentation quality in complex congenital heart disease in humans [22]. The UNet3+ model consists of an advanced encoder-decoder network with deep supervision, designed to enhance multi-scale feature integration, shown in Fig. 1. The network takes each 2D image independently as input and predicts the corresponding 2D segmentation LV blood pool and myocardial masks. The network features two layers per block, five scales, and batch normalization between layers. Final predicted labels are obtained by assigning each pixel to the class with the highest probability.

To reduce the risk of overfitting and improve model generalizability, we applied on-the-fly data augmentation to each 2D image-mask pair during training, using the Python Albumentations library [23]. Augmentation included the following: (i) Horizontal flips (p = 0.5); (ii) 90° rotations (p = 0.5); (iii) Affine transformations with controlled perturbations in scale (±10%), rotation (±10°), and shift (±6.25%); (iv) Brightness adjustments (limit = 0.17); (v) Gamma adjustments (range = 70 to 120); (vi) Contrast-limited adaptive histogram equalization (CLAHE, clip limit = 2.0); and (vii) Gaussian noise (variance range = 30 to 100).

We trained the model with an early stopping criterion (defined as no decrease in validation loss for 25 consecutive epochs), using the Adam optimizer with a batch size of 16 and a Dice loss. All training was performed using TensorFlow on a Linux Workstation (with NVIDIA GeForce RTX 3090).

The dataset was split into 90/15/25 mice for training/validation/testing, respectively (phenotype split shown in Supplementary Information 1). The timeframes which contained ground-truth manual segmentations were extracted, resulting in 224/30/50 SAX stack image-mask pairs, respectively. All timeframes which contained inflow-related artifacts were placed in the training dataset, as these timepoints are not used to calculate clinical volumes and mass. All 2D slices from these SAX stacks were extracted as inputs to the model, resulting in 2087/286/460 2D image-mask pairs for training/validation/testing. Slices beyond the base/apex of the heart that did not contain any blood pool or myocardial segmentations were labeled as background. The images were normalized to standardize intensity values (within the 0 to 1 range) before inputting to the model, with one-hot encoding used to convert the masks to categorical data with three channels representing; background, LV myocardium, and LV blood pool.

The model training code and final weights are available at: https://github.com/mrphys/Open-Source_Pre-Clinical_Segmentation.git.

### 2.3 DL segmentation model evaluation

#### 2.3.1 Test datasets

The DL segmentation model was evaluated on the internal test dataset (acquired at 9.4T) against the ground-truth manual segmentations (460 2D images, taken from all slices of the SAX stack at ED and ES, from the 25 internal test mice, described above).

In addition, we evaluated the DL segmentation model in two external mouse datasets: i) 15 mice acquired on a 7T small-animal Agilent MRI system (Agilent Technologies, Santa Clara, CA, USA) at the University of Oxford, which acquired data with a 4-chanel surface receive array coil; and ii) 10 mice acquired on a 11.7T small-animal Bruker magnetic resonance imaging system (Bruker BioSpin GmbH & Co. KG, Ettlingen, Germany) at the University of Oxford, which acquired data with a 256×256 matrix, without the use of image interpolation. The full imaging protocols for the external datasets can be found in Supplementary Information 2 and Supplementary Information 3, respectively. Ground-truth manual segmentation of the LV blood pool and myocardium was performed as described above. The same observer as used for the internal 9.4T test dataset (WNH, 2 years’ experience), segmented all slices of the SAXs at ED and ES, resulting in 198 2D images for the 7T dataset and 129 2D images for the 11.7T dataset.

All experiments were conducted in full compliance with the European Union Directive 2010/63/European Union and the UK Animals Scientific Procedure Act 1986. Experimental protocols were approved by the UK Home Office under Project License PPL PP9434487 and PPL 30/2278, for the 7T and 11.7T datasets, respectively. These external datasets are not made publicly available.

#### 2.3.2 Model evaluation

Prior to evaluation, only the largest connected component was kept, as identified using connected component labeling and centroid-based tracking, for both blood pool and myocardial classes. This post-processing step was used to remove misclassified ‘islands’ from the segmentations.

The accuracy of the DL segmentation model was quantified using Dice score. As well as overall Dice, we computed per-class Dice and ED and ES Dice, allowing assessment of the interaction between Dice errors in cardiac structure and cardiac phase. Additionally, DL-derived end-diastolic volume (EDV) and end-systolic volume (ESV) were calculated per mouse by summing the labeled blood pool voxels (scaled by voxel volume) across all slices from ED and ES timeframes, respectively. Ejection fraction (EF) was calculated as (EDV-ESV)/EDV, and stroke volume (SV) as EDV-ESV. Myocardial mass was calculated at ED and ES by calculating the sum of the labeled myocardial voxels (scaled by voxel volume and myocardial density – 1.05 g/mL). We also investigated the relationship between Dice score and errors in clinical metrics (EDV, ESV, EF, SV, and myocardial masses).

#### 2.3.3 Inter-observer variability

A secondary observer (ED, 6 years' experience) performed manual segmentation of the LV blood pool and myocardium, at end-diastole and end-systole, for all mice in the internal 9.4T test dataset (n = 25). This enabled assessment of inter-observer variability and provided a benchmark for evaluating the deep learning model performance relative to human observer agreement, in terms of clinical metrics (EDV, ESV, EF, SV, and myocardial masses).

### 2.4 Web-based Inference

We have developed an open-source web-based application, to enable an easy-to-use interface to our DL segmentation model, without the need for local installation. First, an interactive web pipeline was built using *Streamlit*, an open-source Python framework (https://github.com/streamlit/streamlit). The *Streamlit* application is hosted on *Hugging Face*—an open-source platform and ecosystem for building, sharing, and using machine learning models [24]. *Hugging Face* was chosen as their *Spaces* platform provides basic usage with no charge (on a vCPU with 16 GB RAM). The data is encrypted in transit using industry-standard encryption (via TLS/SSL). Uploaded data are processed in-memory (with temporary files deleted after use), and no input data or model outputs are stored.

The *Streamlit* application requires the complete SAX cine image data to be uploaded in NIfTI format, as a zip file using a simple file browser. Pre-processing and DL inference are performed on all 2D images, to generate the segmentation masks. The resulting blood-pool and myocardial volumes are combined across all slices at each timeframe and output in a.csv file. The blood-pool volumes are used to easily identify ED and ES, and these volumes are displayed as a GIF, with the segmentations overlaid.

The web-based inference is available at: https://huggingface.co/spaces/mrphys/Pre-clinical_DL_segmentation.

### 2.5 Statistics

Statistical analyses were performed using the SciPy library (version 1.9.1) in Python, with a *P-value* less than.05 considered statistically significant. Continuous variables are reported as mean ± standard deviation.

The normality of the paired differences between ground-truth manual segmentation and DL segmentations was assessed using the Shapiro-Wilk test. For normally distributed differences, a paired t-test was used, otherwise the Wilcoxon signed-rank test was applied. A two-way repeated measures ANOVA was used to evaluate differences in Dice scores across cardiac timeframes (ED vs ES) and anatomical structures (myocardium vs blood pool).

Agreement between DL-derived and ground-truth-derived clinical metrics was further assessed using Bland–Altman analysis, with manual segmentations used as the clinical standard. Associations between ground-truth and DL-derived clinical metrics, as well as between Dice score and errors in clinical metrics between observers and the DL model were assessed using intraclass correlation coefficients (ICC(2,1)), calculated using a two-way random-effects model.

## 3 Results

Training of our open-source DL segmentation model took ∼4 h (early stopping at 163 epochs). At local inference, the model took 19.6 ± 1.9 ms per 2D image (range: 17.2– 21.8 ms), resulting in 369.4 ± 16.6 ms for all SAX slices at ED and ES. Conventional manual segmentation for all SAX slices at ED and ES takes ∼40 min, therefore the DL segmentation represents ∼x6500 speed-up. Furthermore, our model can perform segmentation of the complete cine stack (all slices of all timeframes) in 4620 ± 676 ms, compared to approximately > 6 h if done manually.

### 3.1 Segmentation accuracy

**Fig. 2:**
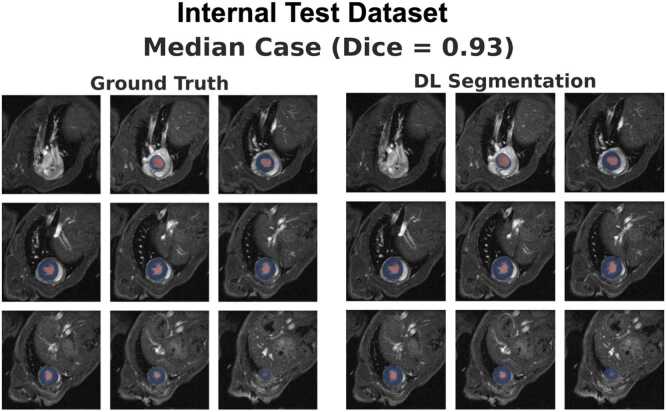
Ground-truth manual segmentations vs. deep learning segmentations for the volume with the median Dice score in the internal 9.4T test dataset. The images show all slices from base-to-apex, with the blood pool segmentation shown in red and the myocardial segmentation in blue. Supplementary Information 4 shows segmentation masks for volumes with best, median, and worst Dice score in the internal 9.4T test dataset (0.96/0.93/0.87, respectively). *DL* deep learning

**Fig. 3:**
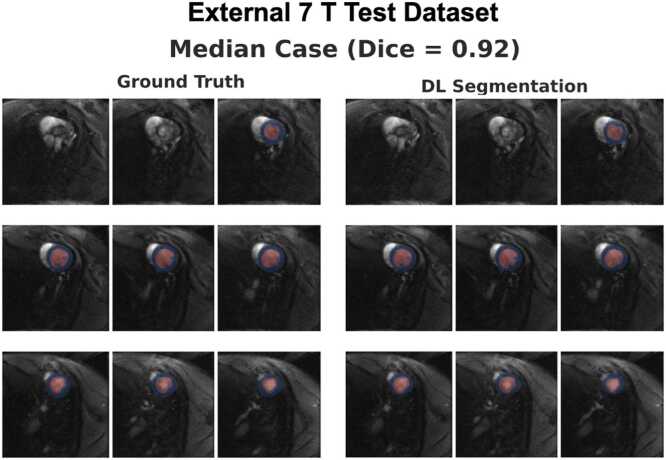
Ground-truth manual segmentations vs. deep learning segmentations for the volume with the median Dice score in the external 7T test dataset. The images show all slices from base-to-apex, with the blood pool segmentation shown in red and the myocardial segmentation in blue. Supplementary Information 5 shows segmentation masks for volumes with best, median, and worst Dice score in the external 7T test dataset (0.94/0.92/0.81, respectively). *DL* deep learning

**Fig. 4:**
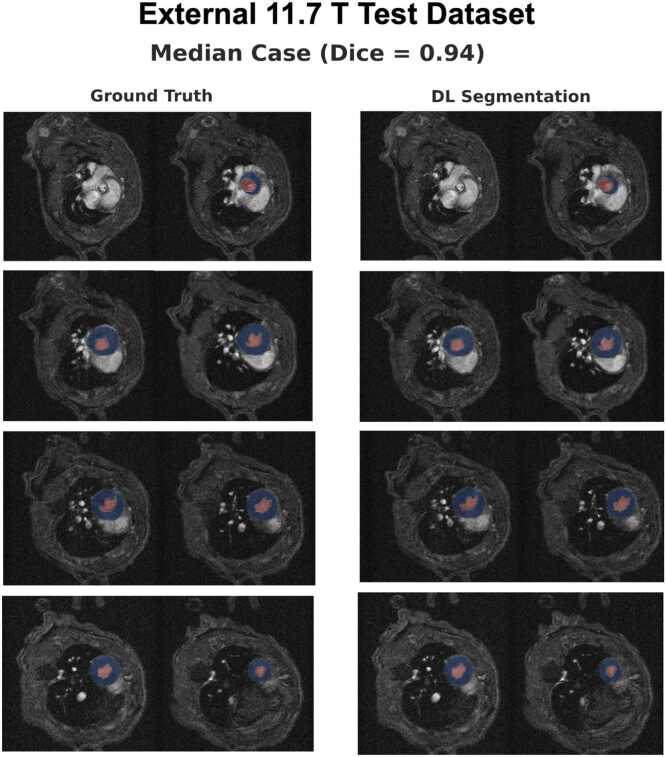
Ground-truth manual segmentations vs. deep learning segmentations for the volume with the median Dice score in the external 11.7T test dataset. The images show all slices from base-to-apex, with the blood pool segmentation shown in red and the myocardial segmentation in blue. Supplementary Information 6 shows segmentation masks for volumes with best, median, and worst Dice score in the external 11.7T test dataset (0.95/0.94/0.91, respectively)

Our open-source DL segmentation model showed strong agreement with ground-truth manual segmentations on all test datasets. Across all segmentation masks, the DL model achieved an average Dice score of 0.92, 0.91 and 0.94, for the internal 9.4T test dataset, the external 7T test dataset, and the external 11.7T test dataset, respectively. Table 1 summarizes per-class (blood pool and myocardium), and cardiac phase-specific (end-diastole and end-systole) Dice scores for all datasets. Fig. 2, Fig. 3 and Fig. 4, show representative images and segmentation masks from the volume with the median Dice score, for the internal 9.4T test dataset, external 7T test dataset, and external 11.7T test dataset, respectively. Supplementary Information 4, Supplementary Information 5 and Supplementary Information 6, show images and segmentation masks for the volumes corresponding to the best, median and worst Dice scores, for the internal 9.4T test dataset, external 7T test dataset and external 11.7T test dataset, respectively.

**Table 1:** Dice scores as calculated between the ground-truth manual segmentation and DL segmentation

	Internal 9.4T Test Dataset	External 7T Test Dataset	External 11.7T Test Dataset
Blood pool – ED	0.95±0.02	0.95±0.02	0.96±0.01
Myocardium – ED	0.89±0.03	0.88±0.04	0.92±0.01
Blood pool – ES	0.93±0.03	0.92±0.04	0.93±0.01
Myocardium – ES	0.92±0.02	0.89±0.03	0.93±0.01
**Overall**	**0.92±0.02**	**0.91±0.03**	**0.94±0.01**

Values shown as mean ± standard deviation. *ED* end diastole, *ES* end systole

It should be noted that there was a small but statistically significant difference in all test datasets between the blood pool and myocardial segmentation accuracy (p < 0.05) and a significant interaction between cardiac phase and structure (p < 0.05). Specifically, the DL segmentation model was more accurate for the blood pool than the myocardium in all test datasets (Dice: ≥ 0.92 for blood pool vs. ≥ 0.88 for myocardium, p < 0.001). There were no statistical differences between ED and ES in any test dataset (Dice: ≥ 0.92 at ED vs. > 0.88 at ES, p > 0.05), however, there was a trend towards significance in the 11.7T dataset. Supplementary Information 7 shows all interaction results.

### 3.2 Cardiac function accuracy

Clinical cardiac volume and function metrics measured from ground-truth manual segmentations and from the DL segmentations for all test datasets are shown in Table 2.

**Table 2:** Clinical metrics extracted from ground-truth manual segmentations and DL segmentations

	Ground-truth segmentation	DL segmentation	Bias (Limits of Agreement)	p-value	ICC [95% CI]
*Internal 9.4T Test Dataset*					
EDV (µL)	65.6±10.0	66.2±10.1	0.6 (−6.0 to 7.3)	0.365	0.94 [0.88, 0.97]
ESV (µL)	27.3±10.1	27.7±10.7	0.4 (−3.2 to 3.9)	0.330	0.98 [0.97, 0.99]
SV (µL)	38.3±6.3	38.5±8.1	0.3 (−6.4 to 6.9)	0.697	0.89 [0.77, 0.95]
EF (%)	59.3±10.7	59.1±12.3	-0.2 (−5.7 to 5.3)	0.699	0.97 [0.93, 0.99]
Myocardial Mass at ED (mg)	105.2±26.8	107.5±25.5	2.3 (−12.8 to 17.4)	0.165	0.95 [0.90, 0.98]
Myocardial Mass at ES (mg)	111.2±25.6	112.8±26.1	1.6 (−13.0 to 16.3)	0.298	0.96 [0.91, 0.98]
*External 7T Test Dataset*					
EDV (µL)	56.5±12.5	56.7±13.1	0.2 (−3.8 to 4.2)	0.743	0.99 [0.96, 1.00]
ESV (µL)	20.7±6.2	20.9±5.9	0.2 (−3.8 to 4.1)	0.735	0.95 [0.84, 0.98]
SV (µL)	35.8±8.3	35.8±8.7	0.0 (−4.2 to 4.2)	0.995	0.97 [0.91, 0.99]
EF (%)	63.6±5.5	63.3±5.6	-0.2 (−6.3 to 5.9)	0.782	0.85 [0.60, 0.94]
Myocardial Mass at ED (mg)	82.2±20.6	77.4±17.2	-4.8 (−22.1 to 12.4)	0.060	0.87 [0.71, 0.96]
Myocardial Mass at ES (mg)	77.7±21.2	79.1±17.8	1.3 (−14.8 to 17.5)	0.562	0.91 [0.76, 0.97]
*External 11.7T Test Dataset*					
EDV (µL)	60.8±11.4	60.7±11.5	-0.1 (−2.5 to 2.3)	0.105	0.99 [0.98, 1.00]
ESV (µL)	24.8±7.1	23.6±7.1*	-1.3 (−3.4 to 0.9)	0.007	0.97 [0.95, 1.00]
SV (µL)	36.0±6.3	37.1±6.8*	1.2 (−1.2 to 3.5)	0.017	0.97 [0.94, 1.00]
EF (%)	59.6±6.0	61.6±6.4*	2.0 (−1.3 to 5.3)	0.006	0.92 [0.86, 0.99]
Myocardial Mass at ED (mg)	90.7±11.3	92.5±11.8*	1.8 (−5.4 to 9.0)	0.177	0.95 [0.80, 0.98]
Myocardial Mass at ES (mg)	98.4±12.9	99.6±11.3	1.2 (−11.0 to 13.4)	0.577	0.88 [0.56, 0.97]

Values shown as mean ± standard deviation. Bias and limits of agreement from Bland–Altman analysis.

*EDV* end diastolic volume, *ESV* end systolic volume, etc.

* shows statistically significant difference between ground-truth manual segmentation-derived metric and DL segmentation-derived metric, p < 0.05

**Fig. 5:**
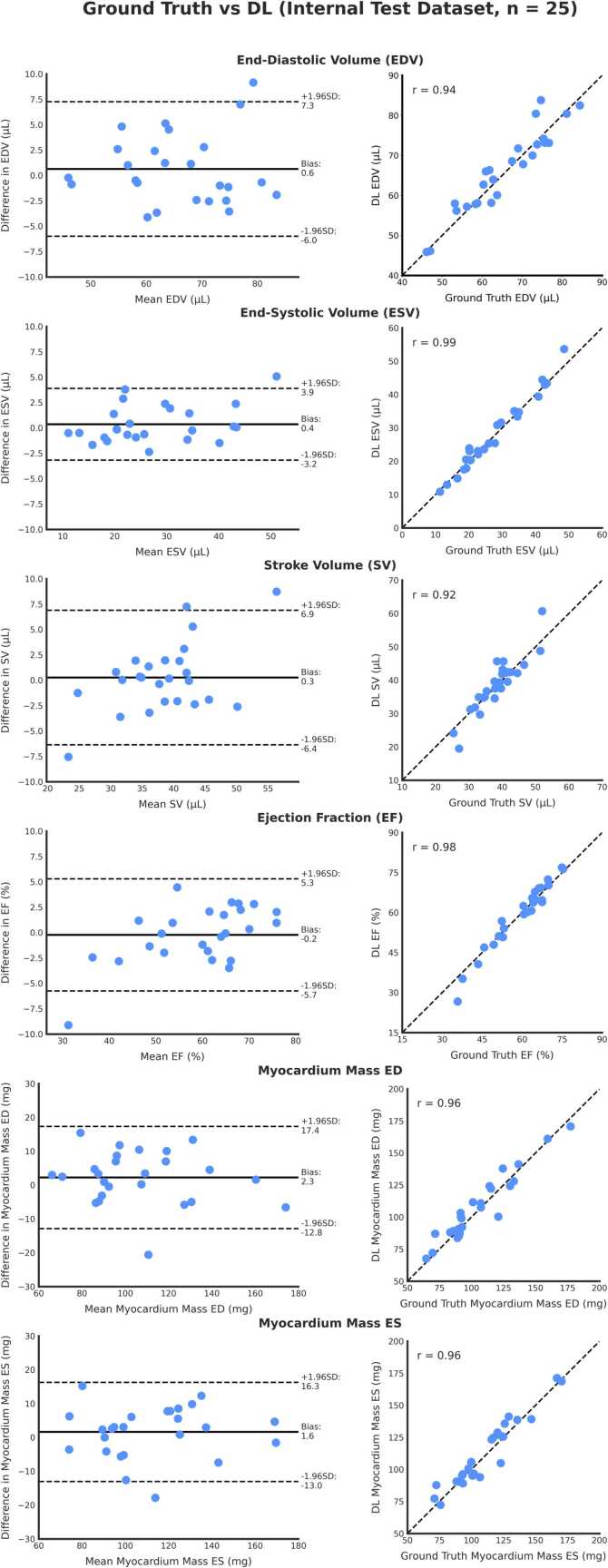
Bland–Altman plots (left) and line of equality plots (right) for the internal 9.4T test dataset comparing ground-truth manual vs. DL-derived functional metrics; EDV, ESV, SV, EF, and myocardial mass at ED and ES (n = 25). *DL* Deep learning, *EDV* End-diastolic volume, *ESV* End-systolic volume, *SV* Stroke volume, *EF* Ejection fraction, *ES* End-systole, *ED* End-diastole

**Fig. 6:**
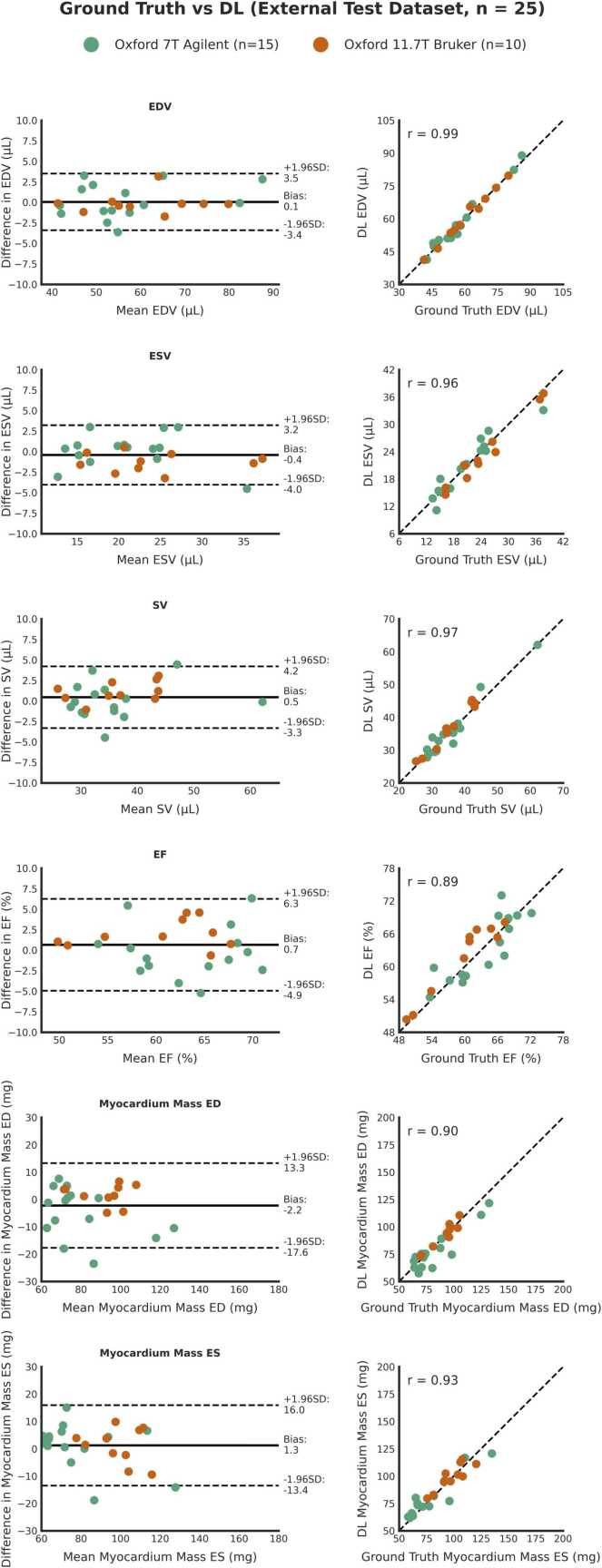
Bland–Altman plots (left) and line of equality plots (right) for the external test datasets (7T and 11.7T, combined), comparing ground-truth manual vs. DL-derived functional metrics; EDV, ESV, SV, EF, and myocardial mass at ED and ES (n = 25). *DL* Deep learning, *EDV* End-diastolic volume, *ESV* End-systolic volume, *SV* Stroke volume, *EF* Ejection fraction, *ES* End-systole, *ED* End-diastole

Bland–Altman and correlation plots for the internal 9.4T test dataset are shown in Fig. 5, and for the external test datasets (7T and 11.7T) are shown in Fig. 6. There was good agreement between clinical metrics from ground-truth manual segmentations and DL segmentations in all test datasets, in terms of EDV (ICC ≥ 0.94), ESV (ICCs ≥ 0.95), SV (ICC ≥ 0.89), EF (ICC ≥ 0.85), and myocardial mass at both ED ( r ≥ 0.87) and ES (ICC ≥ 0.88). The only statistically significant differences between clinical metrics from ground-truth manual segmentations and DL segmentations were found in the 11.7T external test dataset, with slightly lower ESV (23.6 ± 7.1 vs. 24.8 ± 7.1, respectively. p = 0.007), resulting in slightly higher SV (37.1 ± 6.8 vs. 36.0 ± 6.3, respectively. p = 0.017), slightly higher EF (61.6 ± 6.4 vs. 59.6 ± 6.0, respectively, p = 0.006).

A negative correlation was found between blood pool Dice and volume errors in all test datasets, with stronger correlation for EDV (r ≤ −0.61) than ESV (r ≤ −0.45). Similarly, a negative correlation was also found between myocardial Dice and myocardial mass errors for all test datasets, with stronger correlation for ED (r ≤ −0.61) than ES (r ≤ −0.25). All associations reached statistical significance, except myocardial Dice and myocardial mass errors at ES for the 11.7T test dataset. All association results are summarized in Supplementary Information 8.

## 4 Inter-observer variability

Bland–Altman analysis and ICC(2,1) demonstrated good agreement between the DL model and both human observers across all clinical metrics on the internal 9.4T test dataset (n = 25). Full results shown in Supplementary Information 9. For EDV, ESV, SV, and EF, the biases were small and centered near zero, with narrow limits of agreement comparable to inter-observer variability. Corresponding ICCs were high for DL vs. primary observer (0.89–0.98), DL vs. secondary observer (0.85–0.96), and between observer 1 and observer 2 (0.88–0.96), indicating good reliability. Overall, the DL model performed comparably to a secondary human observer, with agreement metrics consistently within the range of human inter-observer variability.

### 4.1 Time-volume curves

**Fig. 7:**
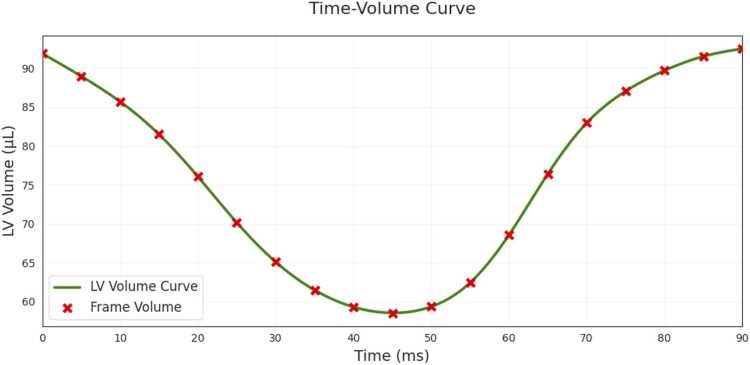
An example LV time-volume curve from one mouse in the internal 9.4T test dataset, which can be easily extracted from the DL segmentation model applied to all timeframes and all slices. *LV* left ventricular, *DL* deep learning

Although not necessary for this study, the DL segmentation model can be applied to all 2D slices of all timeframes of the SAX cine to enable rapid calculation of time-volume curves, as shown in Fig. 7. These curves can be seen to be smooth and can be used to easily extract EDV (maximum of the time-volume curve) and ESV (minimum of the time-volume curve), as is performed in the web-based application.

## 5 Web-based inference

**Fig. 8:**
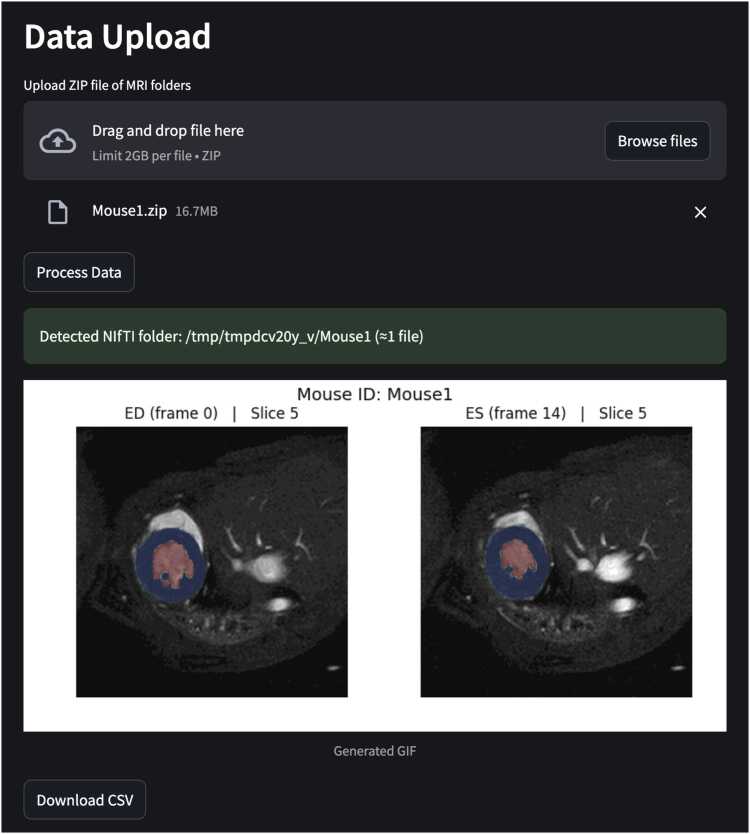
The web-based inference application. The user uploads a zip file containing their NIfTI files, and then selects “Process Data”. When the application has finished GIFs of the imaging stacks at ED and ES are shown with the segmentations overlaid (blood pool in red and myocardium in blue). The application also enables the user to download a.csv file containing the blood pool and myocardial volumes at each time frame

The *Hugging Face Spaces* platform enables straightforward upload of pre-clinical CMR data. For a complete cine short-axis stack (∼200 2D images encompassing all slices and cardiac timeframes), the complete application takes ∼3–4 min. It accurately identifies ED and ES, and generates left ventricular blood pool and myocardial volumes which are consistent with those obtained from the native Python implementation. In addition, the platform provides visual overlays of the segmentation masks on the cine images, offering users an intuitive means of assessing segmentation quality (Fig. 8).

## 6 Discussion

This paper presents the first publicly available pre-clinical CMR dataset with expert manual segmentations, together with the first open-source deep learning model for cardiac segmentation in mice. By providing standardized data, benchmark segmentations, and an easily accessible inference tool, this work addresses a key bottleneck in pre-clinical cardiovascular research, namely the lack of reproducible and comparable resources for automated image analysis.

Beyond demonstrating that our DL model achieves high segmentation accuracy, we have shown that the DL-derived ventricular volumes, functional parameters, and myocardial mass are consistent with manual ground-truth analysis and fall within the range of inter-observer variability. Furthermore, evaluation of the DL model on two independent external datasets (acquired from a second site, at different field strengths, with different protocols and with a different scanner vendor) demonstrated consistent segmentation performance and agreement of derived clinical metrics, suggesting that the proposed model may be robust approach across different imaging environments.

However, small systematic differences between ground-truth manual segmentation and DL segmentations were observed, including lower Dice in the myocardium than in the blood pool. This bias seems to be from ambiguity in the basal and apical slices rather than mid-ventricular regions, where partial volume effects and subjective criteria for basal slice inclusion introduce variability. Importantly, the magnitude of these differences was comparable to human inter-observer variability, indicating that they primarily reflect intrinsic ambiguities in pre-clinical CMR. Improvements in spatial resolution, standardized slice planning, and physiological gating to reduce artifacts would be expected to reduce these discrepancies.

At inference, the DL model was able to segment the LV blood pool and myocardium, on all slices and all timeframes of a SAX cine dataset in <4.6 s. This enables rapid calculation of smooth LV time-volume curves, allowing extraction of other metrics, including peak ejection rate and peak filling rate, which can provide additional insight into ventricular function [25], [26], as well as wall thickness measurements to further enhance clinical assessments.

## 7 Comparison with previous studies

While DL-based segmentation is increasingly used in human cardiac MRI, direct translation of these models to pre-clinical imaging is not feasible. Differences in cardiac anatomy, heart size, heart rate, and image appearance across species, combined with variations in imaging protocols, spatial resolution, and contrast mechanisms, introduce substantial domain shifts which DL models are sensitive to. This limitation has been demonstrated even between closely related species; for example, models trained on rhesus macaque CMR data have been shown to perform poorly when applied to baboon CMR, despite similar cardiac anatomy, underscoring the need for species-specific segmentation approaches [27].

Several studies have explored DL-based cardiac segmentation in mice, however, they relied on small, private cohorts and limited validation strategies. This is in part attributable to the substantial effort required to acquire and segment high-resolution pre-clinical CMR in large numbers of animals. Xu et al. [14] employed a 2D UNet with leave-one-out validation on 12 mouse datasets, reporting Dice coefficients of 0.89 for the LV blood pool and 0.84 for the myocardium. Hammouda et al. [15] reported higher Dice scores (0.96 for LV blood pool and 0.93 for myocardium) using a 2D fully convolutional network, however, these results were derived from only six mouse datasets. Zufiria et al. [16] proposed a 3D multi-label network trained on 21 mice for bi-ventricular segmentation, however, they reported lower Dice scores for LV blood pool (0.85) and myocardium (0.81).

DL-based cardiac segmentation has also been investigated in rat MRI, where substantially larger training datasets are available. This has enabled the use of 3D networks, which may improve segmentation accuracy at the base and apex of the heart. Fernández-Llaneza et al. [10] and Gondova et al. [11] have trained 3D UNets on >500 rat datasets to perform LV blood pool segmentation only, reporting Dice coefficients of >0.93. The Dice scores achieved in the present study are of comparable magnitude for LV blood pool segmentation to those rat studies, while additionally providing robust myocardial segmentation in a more challenging imaging regime characterized by thinner myocardial walls, higher heart rates, and reduced signal-to-noise ratio.

## 8 Limitations

The main limitation of this study is that ground-truth manual segmentations were performed by a single operator. This was done to ensure consistency of segmentation, but could result in biases in DL models. However, comparison with a second observer on the internal test dataset (n = 25 mice) demonstrated good agreement, partially mitigating this concern. Additionally, our publicly available dataset is from a single center which may lead to bias. However, we demonstrated consistent segmentation performance on two independent external datasets acquired at a separate site, at different field strengths (7T and 11.7T), a different vendor and different imaging protocols. However, further validation on multi-center datasets would be required to fully establish generalizability. On this point, although we did test various protocols, all our test data were prospectively gated data, thus further work is required to assess its robustness on retrospectively gated data. Finally, in this paper, we only consider segmentations for the LV blood pool and myocardium. Automated RV segmentation is likely to be more challenging due to the thin myocardial wall, complex geometry, and less well-defined boundaries, particularly given the limited spatial resolution and signal-to-noise ratio of pre-clinical imaging. However, extension of the framework to right ventricular structures represents an important direction for future work.

## 9 Conclusion

To conclude, we present the first publicly available pre-clinical CMR dataset, as well as the first open-source deep learning model for cardiac segmentation in mice. In addition, we have provided an open-source, simple-to-use, free-to-use web-based application to perform inference using our model. We demonstrated that DL-derived clinical measurements were comparable to human inter-observer variability, and that the DL model performed consistently across independent external datasets acquired at different field strengths, supporting the robustness of the proposed approach. We believe that these tools are instrumental in driving advancements and innovations in pre-clinical CMR, by enabling independent validation and benchmarking of models. We hope this will lead to widespread integration of DL segmentation tools in pre-clinical CMR, increasing reproducibility, speeding-up post-processing and aiding in the development of novel therapies for cardiovascular disease.

## Author contributions

**Wan Shah: Writing – review & editing, Software, Methodology,** Formal analysis, Data curation. **Daniel J. Stuckey:** Writing – review & editing, Supervision, Resources, Project administration, Methodology, Investigation, Funding acquisition, Formal analysis, Data curation, Conceptualization. **Tina Yao:** Writing – review & editing, Visualization, Validation, Software, Methodology. **Mark Wrobel:** Writing – review & editing, Software, Methodology. **Emily Deniszczyc:** Formal analysis, Writing – review & editing. **Zhiping Feng:** Data curation, Writing – review & editing. **Stephanie Anderson:** Data curation, Resources, Writing – review & editing. **Ruaraidh Campbell:** Writing – review & editing, Software, Methodology. **Vivek Muthurangu:** Writing – review & editing, Writing – original draft, Validation, Supervision, Software, Project administration, Methodology, Formal analysis, Data curation. **Jennifer Steeden:** Writing – review & editing, Writing – original draft, Validation, Supervision, Software, Methodology, Formal analysis, Data curation, Conceptualization.

## Declaration of competing interests

The authors declare that they have no known competing financial interests or personal relationships that could have appeared to influence the work reported in this paper.
