# Supplementary material for: Open-source pre-clinical image segmentation: mouse cardiac magnetic resonance imaging datasets with a deep learning segmentation framework
Source: J Cardiovasc Magn Reson. 2026 Feb 17;28(1):102706. doi: 10.1016/j.jocmr.2026.102706 (PMC13237537; doi:10.1016/j.jocmr.2026.102706)
Supplement: Supplementary file 1 — Supplementary material [file mmc1.docx]

**SUPPLEMENTARY INFORMATION**

**Supplementary Information 1:**

Myocardial phenotypes in the publicly-available 9.4 T dataset, and the split for training/validation/test used in our open-source DL segmentation model.

| **Phenotype** | **Overall (n=130)** | **Training (n=90)** | **Validation (n=15)** | **Test (n=25)** |
| --- | --- | --- | --- | --- |
| Control | 39 | 27 | 5 | 7 |
| Myocardial infarction with grafting of a biomaterial patch | 11 | 11 | 0 | 0 |
| Transgenic model of dilated cardiomyopathy | 51 | 29 | 8 | 14 |
| Transverse aortic constriction model | 29 | 23 | 2 | 4 |

**Supplementary Information 2:**

The external 7 T dataset includes 15 mice, imaged at the University of Oxford between October 2020 and June 2021. Each mouse consists of a complete short-axis cine stack, with ~8 slices (range 7–9) and ~19 cardiac timeframes (range 15–25), resulting in a total of 2,271 2D images. The dataset consists of all control female C57/Blk6 mice (16-24 weeks old).

All experiments were conducted in full compliance with the European Union Directive 2010/63/European Union and the UK Animals Scientific Procedure Act 1986. Experimental protocols were approved by the UK Home Office under Project Licenses PPL PP9434487.

The dataset was acquired on a 7 T small-animal Agilent MRI system (Agilent Technologies, Santa Clara, USA) with a 72 mm linearly polarised volume transmit coil and a 4-channel 20 mm surface receive array (Rapid Biomedical GmbH, Germany) with an integrated preamplifier. A small animal physiological monitoring system was used to maintain depth of anaesthesia and animal physiology. Internal temperature was monitored using a rectal thermometer and maintained using warm air. An in-house developed ECG and respiratory gating device was used for monitoring heart and respiration signals, derived from two needles subcutaneously inserted in the front paws. Respiratory signals could also be derived from a loop loosely fitted on the chest and abdomen of the animals.

For each mouse, cardiac-gated, spoiled gradient echo (GRE) cine MRI was acquired as described in [28]. A stack of 6-8 contiguous slices was used to ensure full coverage of the left ventricle. Prospective ECG triggering was used to synchronize image acquisition with the cardiac cycle, and respiratory gating was used to remove breathing motion. The imaging parameters were: slice thickness = 1.0 mm; TE/TR = 1.3/4.6 ms; flip angle = ~30°; acquisition matrix size = 128 × 128; interpolation factor = 2.0; interpolated pixel size = 0.1 × 0.1 mm, signal averages = 4. The temporal resolution was 4.6 ms, resulting in 15-25 timeframes per cardiac cycle (depending on heart rate).

**Supplementary Information 3:**

The external 11.7 T dataset includes 10 mice, imaged at the University of Oxford between June 2007 and October 2007. Each mouse consists of a complete short-axis cine stack, with ~9 slices (range 8–10) and ~21 cardiac timeframes (range 15–27), resulting in a total of 1,834 2D images. The dataset consists of all control male C57/Blk6 mice (16-24 weeks old).

All experiments were conducted in full compliance with the European Union Directive 2010/63/European Union and the UK Animals Scientific Procedure Act 1986. Experimental protocols were approved by the UK Home Office under Project License PPL 30/2278.

The dataset was acquired on an 11.7 T small-animal Bruker MRI system (Bruker BioSpin GmbH & Co. KG, Ettlingen, Germany) comprising a vertical magnet (bore size = 123 mm; Magnex Scientific, Oxon, UK), a Bruker Avance console (Bruker Medical, Ettlingen, Germany), and a shielded gradient system (Magnex Scientific, Oxon, UK). Imaging was acquired using quadrature driven birdcage coils with inner diameters of 40 mm (Rapid Biomedical, Wurzburg, Germany). A small animal physiological monitoring system was used to maintain depth of anaesthesia and animal physiology. Internal temperature was monitored using a rectal thermometer and maintained using warm air. An in-house developed ECG and respiratory gating device was used for monitoring heart and respiration signals, derived from two needles subcutaneously inserted in the front paws. Respiratory signals could also be derived from a loop loosely fitted on the chest and abdomen of the animals. Animals were anaesthetised under a mixture of 2% isoflurane in oxygen.

For each mouse, cardiac-gated, spoiled gradient echo (GRE) cine MRI was acquired. A stack of 7-9 contiguous slices was used to ensure full coverage of the left ventricle. Prospective ECG triggering was used to synchronize image acquisition with the cardiac cycle, and respiratory gating was used to remove breathing motion. The imaging parameters were: slice thickness = 1.0 mm; TE/TR = 1.43/4.6 ms; flip angle = ~15°; acquisition matrix size = 256 × 256; interpolation factor = 1.0; pixel size = 0.1 × 0.1 mm, signal averages = 2. The temporal resolution was 4.6 ms, resulting in 15-27 timeframes per cardiac cycle (depending on heart rate).

**Supplementary Information 4:**

Ground-truth manual segmentations vs. deep learning segmentations for the volumes with the best, median, and worst overall Dice score in the internal 9.4 T test dataset. The images show all slices from base-to-apex, with the blood pool segmentation shown in red and the myocardial segmentation in blue.

**
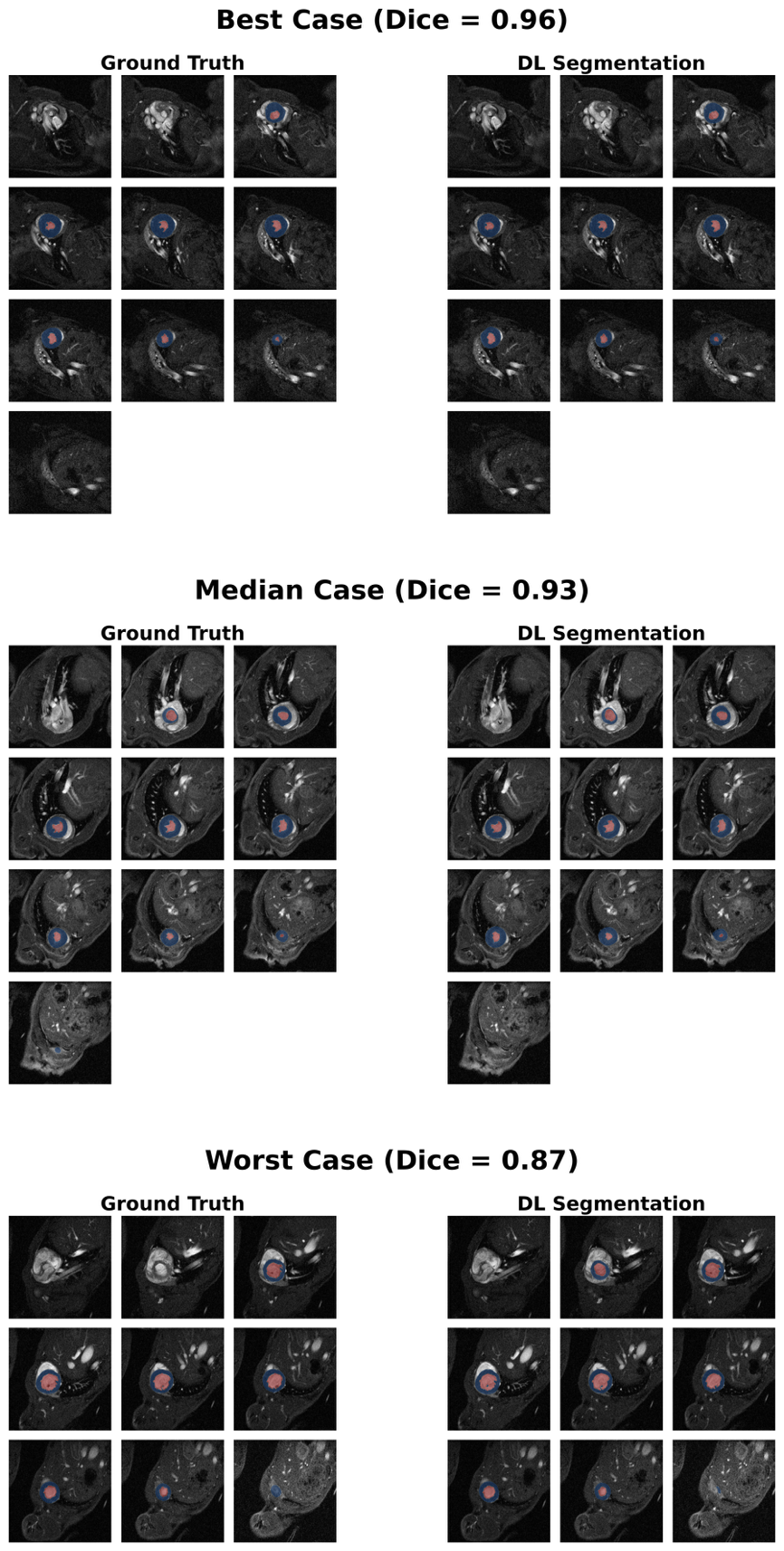
**

**Supplementary Information 5:**

Ground-truth manual segmentations vs. deep learning segmentations for the volumes with the best, median, and worst overall Dice score in the external 7 T test dataset. The images show all slices from base-to-apex, with the blood pool segmentation shown in red and the myocardial segmentation in blue.

**
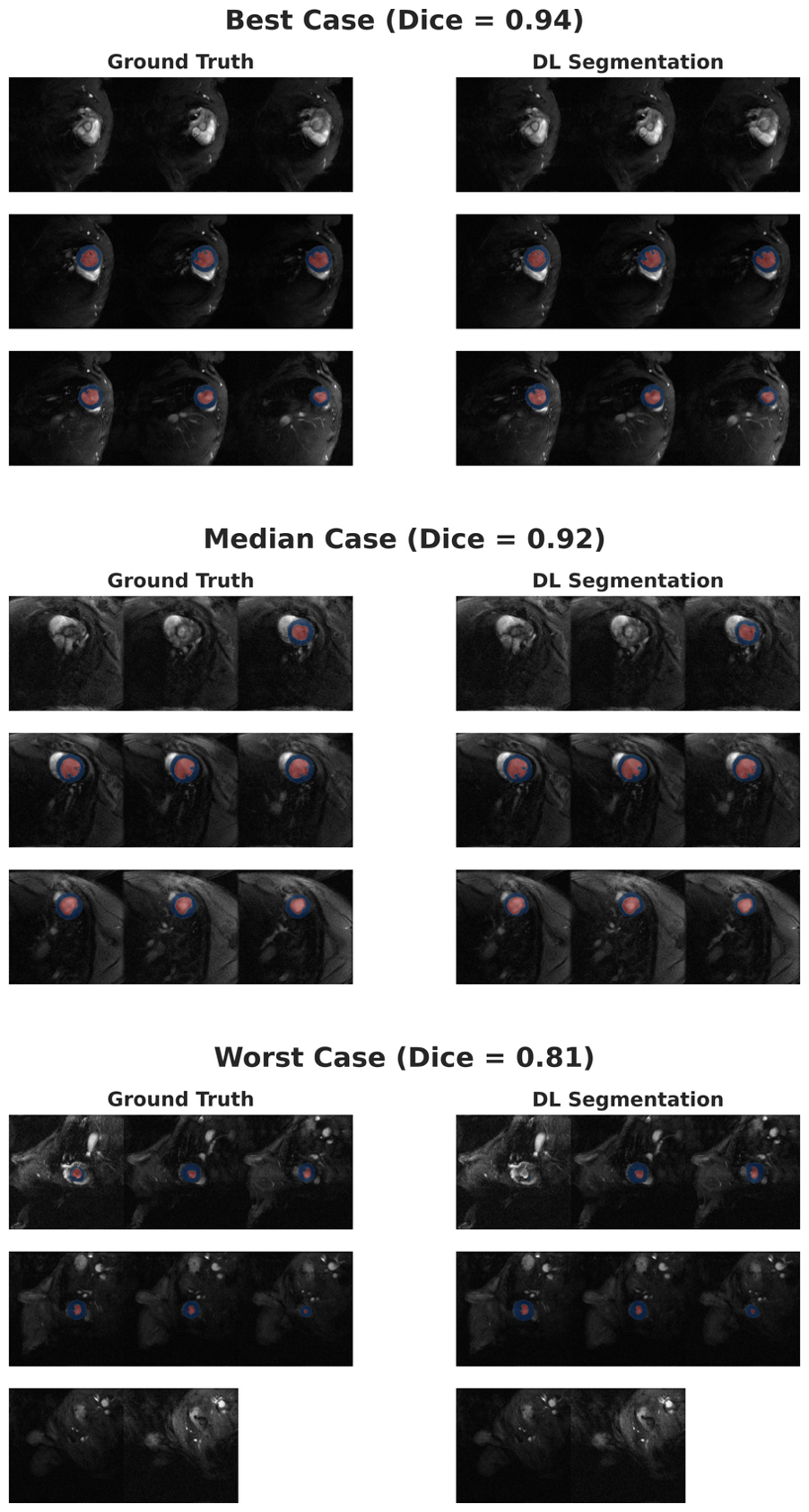
**

**Supplementary Information 6:**

Ground-truth manual segmentations vs. deep learning segmentations for the volumes with the best, median, and worst overall Dice score in the external 11.7 T test dataset. The images show all slices from base-to-apex, with the blood pool segmentation shown in red and the myocardial segmentation in blue.

**
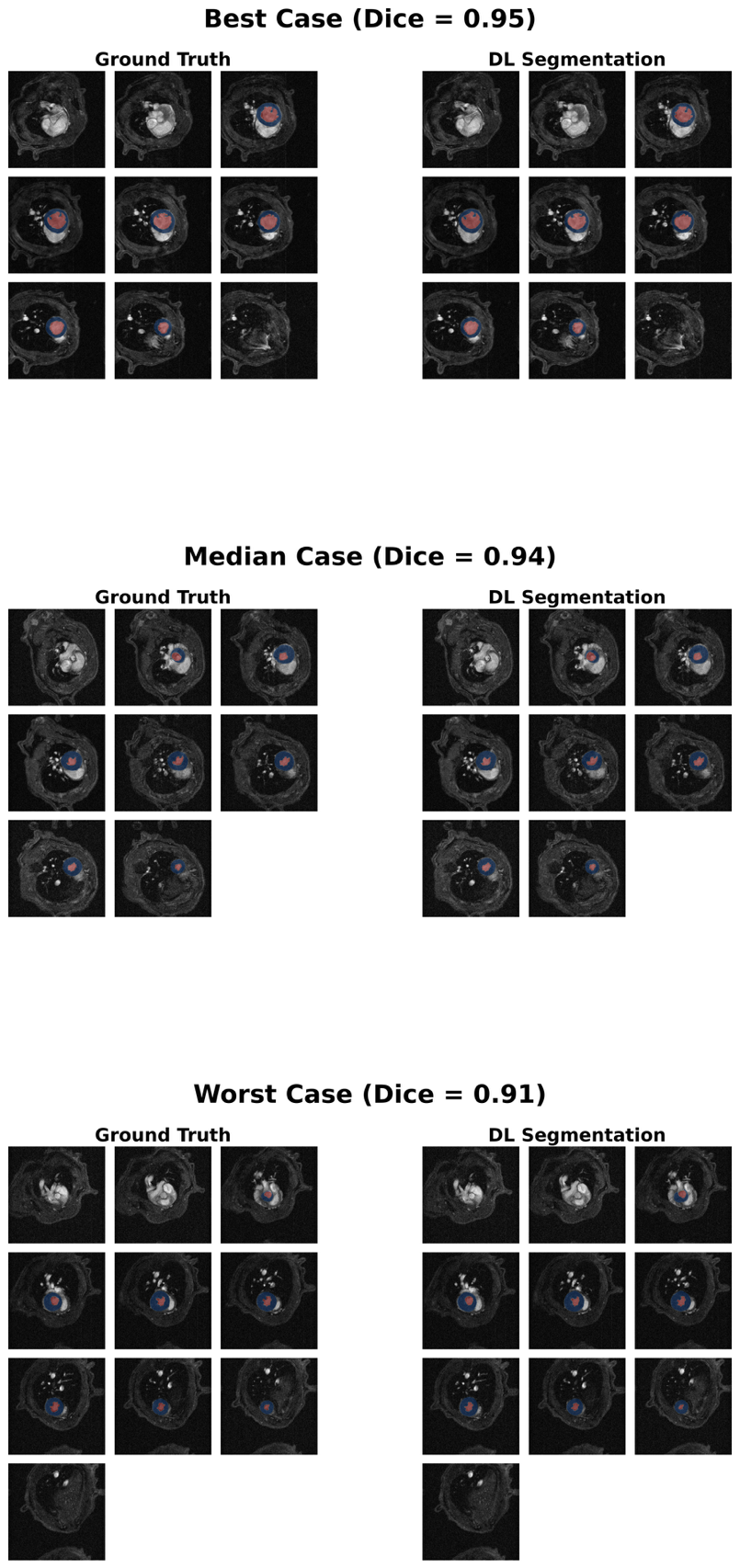
**

**Supplementary Information 7:**

Results from the two-way repeated measures ANOVA, used to evaluate differences in Dice scores across cardiac timeframes (ED vs. ES) and anatomical structures (myocardium vs. blood pool), as well as interaction between Dice scores in cardiac structure and cardiac phase.

| **Effect** |  | **p-value** |  |
| --- | --- | --- | --- |
| **Internal 9.4 T Test Dataset** | | | |
| Cardiac Phase: ED vs. ES |  | 0.6515 |  |
| Structure: Blood pool vs. Myocardium |  | <0.001 |  |
| Interaction: Cardiac Phase x Structure |  | <0.001 |  |
| **External 7 T Test Dataset** | | | |
| Cardiac Phase: ED vs. ES |  | 0.3330 |  |
| Structure: Blood pool vs. Myocardium |  | <0.001 |  |
| Interaction: Cardiac Phase x Structure |  | 0.0004 |  |
| **External 11.7 T Test Dataset** | | | |
| Cardiac Phase: ED vs. ES |  | 0.0531 |  |
| Structure: Blood pool vs. Myocardium |  | 0.0001 |  |
| Interaction: Cardiac Phase x Structure |  | <0.001 |  |

**Supplementary Information 8:**

Associations results between Dice score and errors in clinical metrics as tested using Pearson correlation coefficients.

| **Structure-Phase** | **r** | **p-value** |
| --- | --- | --- |
| **Internal 9.4 T Test Dataset** | | |
| Blood pool - ED | -0.61 | 0.001 |
| Myocardium - ED | -0.61 | 0.001 |
| Blood pool – ES | -0.45 | 0.023 |
| Myocardium - ES | -0.50 | 0.012 |
| **External 7 T Test Dataset** | | |
| Blood pool - ED | -0.64 | 0.011 |
| Myocardium - ED | -0.81 | <0.001 |
| Blood pool – ES | -0.54 | 0.038 |
| Myocardium - ES | -0.63 | 0.011 |
| **External 11.7 T Test Dataset** | | |
| Blood pool - ED | -0.90 | <0.001 |
| Myocardium - ED | -0.88 | <0.001 |
| Blood pool – ES | -0.69 | 0.027 |
| Myocardium - ES | -0.25 | 0.493 |

**Supplementary Information 9:**

Bland-Altman analysis and intraclass correlation coefficients (ICC(2,1)) demonstrating agreement of derived clinical metrics from the primary observer (O1), secondary observer (O2), and DL model, on the internal 9.4 T test dataset.

|  | **Bias (Limits of Agreement)** | | | **ICC** **[95% CI]** |
| --- | --- | --- | --- | --- |
| **EDV (µL)** | | |  | |
| DL vs. O1 | 0.64 (-5.99 to 7.27) | | | 0.94 [0.89 - 0.97] |
| O2 vs. O1 | -0.89 (-8.32 to 6.53) | | | 0.92 [0.84 - 0.95] |
| DL vs. O2 | 1.53 (-6.18 to 9.24) | | | 0.91 [0.81 - 0.96] |
| **ESV (µL)** | | |  | |
| DL vs. O1 | 0.37 (-3.17 to 3.90) | | | 0.98 [0.97 - 0.99] |
| O2 vs. O1 | 0.76 (-4.33 to 5.85) | | | 0.96 [0.92 - 0.99] |
| DL vs. O2 | -0.40 (-6.10 to 5.30) | | | 0.96 [0.93 - 0.98] |
| **SV (µL)** | |  | | |
| DL vs. O1 | 0.27 (-6.35 to 6.90) | | | 0.89 [0.77 - 0.95] |
| O2 vs. O1 | -1.66 (-6.97 to 3.65) | | | 0.88 [0.75 - 0.94] |
| DL vs. O2 | 1.93 (-5.15 to 9.01) | | | 0.85 [0.71 - 0.93] |
| **EF (%)** |  | | | |
| DL vs. O1 | -0.23 (-5.75 to 5.30) | | | 0.97 [0.93 - 0.99] |
| O2 vs. O1 | -1.77 (-6.89 to 3.35) | | | 0.96 [0.91 - 0.98] |
| DL vs. O2 | 1.54 (-5.01 to 8.10) | | | 0.95 [0.90 - 0.98] |
| **Myocardium Mass - ED (mg)** | | | | |
| DL vs. O1 | 2.25 (-12.84 to 17.35) | | | 0.95 [0.90 - 0.98] |
| O2 vs. O1 | 7.37 (-13.56 to 28.30) | | | 0.90 [0.79 - 0.96] |
| DL vs. O2 | -5.11 (-25.06 to 14.83) | | | 0.92 [0.84 - 0.96] |
| **Myocardium Mass - ES (mg)** | | | | |
| DL vs. O1 | 1.62 (-13.04 to 16.29) | | | 0.96 [0.91 - 0.98] |
| O2 vs. O1 | 7.03 (-9.92 to 23.97) | | | 0.92 [0.82 - 0.96] |
| DL vs. O2 | -5.40 (-20.79 to 9.99) | | | 0.94 [0.88 - 0.97] |
